# Clinical course of epilepsy and white matter abnormality linked to a novel *DYRK1A* variant

**DOI:** 10.1038/s41439-021-00157-7

**Published:** 2021-07-12

**Authors:** Tetsuya Okazaki, Hiroyuki Yamada, Kaori Matsuura, Noriko Kasagi, Noriko Miyake, Naomichi Matsumoto, Kaori Adachi, Eiji Nanba, Yoshihiro Maegaki

**Affiliations:** 1 Division of Clinical Genetics, Tottori University Hospital, Yonago, Japan; 2 Division of Child Neurology, Department of Brain and Neurosciences, Faculty of Medicine, Tottori University, Yonago, Japan; 3 Department of Fundamental Nursing, School of Health Science, Tottori University Faculty of Medicine, Yonago, Japan; 4 Department of Human Genetics, Yokohama City University Graduate School of Medicine, Yokohama, Japan; 5 Research Initiative Center, Organization for Research Initiative and Promotion, Tottori University, Yonago, Japan; 6 Research Strategy Division, Organization for Research Initiative and Promotion, Tottori University, Yonago, Japan

**Keywords:** Paediatric neurological disorders, Disease genetics

## Abstract

Epilepsy and white matter abnormality have been reported in *DYRK1A*-related intellectual disability syndrome; however, the clinical course has yet to be elucidated. Here, we report the clinical course of an 18-year-old male with a novel heterozygous *DYRK1A* variant (NM_001396.4: c.957C>G, p.Tyr319*); based on previous reports, epilepsy with this syndrome tends to be well controlled. Follow-up MRIs of the patient’s lesion revealed slightly reduced signal intensity compared to the first image.

Dual-specificity tyrosine-phosphorylation-regulated kinase (DYRK) catalyzes the phosphorylation of serine and threonine residues on exogenous substrates, as well as the phosphorylation of tyrosine residues within its own activation loop [1]. The *DYRK1A* gene (NM_001396.4) encodes a member of the DYRK family. *DYRK1A* is located on chromosome 21q22.13, which is in the Down syndrome critical region of the chromosome [2]. The DYRK1A protein is essential for neurogenesis, neuronal differentiation and proliferation, the cell cycle, and synaptic plasticity [1]. Patients with the *DYRK1A* pathogenic variant show “mental retardation, autosomal dominant 7” (MIM #614104), which is also called *DYRK1A*-related intellectual disability syndrome. In addition to intellectual disability, other frequently occurring features include intrauterine growth restriction, feeding difficulties with failure to thrive, microcephaly, seizures, dysmorphic facial features, and developmental delays [2, 3]. Epileptic events and data from magnetic resonance imaging (MRI) assessments have been reported for patients with *DYRK1A*-related intellectual disability syndrome; however, the clinical course is not well understood.

The index patient was an 18-year-old male who had been born to nonconsanguineous parents at 40 weeks and 6 days. The family history included no reports of intellectual disability, developmental delay, or epilepsy. At birth, his weight was 2604 g (−2.2 SD), his body length was 44.5 cm (−2.9 SD), and his head circumference was 30.5 cm (−2.4 SD); there was no birth asphyxia. He underwent palate surgery with a Hotz-type plate at 6 days, 1 month, 2 months, 6 months, and 10 months to repair a cleft palate and lip. At age 11, he underwent cleft palate bone graft surgery. He was able to control his head at the age of 3 months, independently sat at the age of 12 months, and independently walked at the age of 2 years and 3 months. By age 18, he had never uttered any meaningful words. His Tsumori–Inage infant mental development quotient was 20 at the age of 7 years and 12 at the age of 17 years. He was diagnosed with autistic spectrum disorder due to obsessive-compulsive behavior and impairments in social interaction and social communication. He experienced his first febrile seizure at the age of 1 year. At the age of 1 year and 6 months, he had five episodes of febrile seizure, and valproate was initiated. The febrile seizure episodes gradually decreased, disappearing entirely at the age of 3 years and 6 months. At the age of 7 years and 1 month, he discontinued valproate at his parents’ discretion. At 7 years and 2 months, he experienced his first nonfebrile general atonic seizure with cyanosis. After he resumed valproate, his seizure episodes ceased. At 12 years and 2 months, his valproate dose was gradually tapered off, but generalized clonic seizures occurred at the age of 12 years and 8 months. The patient resumed valproate, and his seizure episodes ceased. After 4 years without seizure episodes, valproate was gradually tapered off by age 17. Generalized seizures recurred three months later; accordingly, the same 400 mg dose of valproate was resumed. The patient had undergone interictal electroencephalography (EEG) multiple times since early childhood, but there were no abnormal findings. His first head MRI, taken at the age of 11 years, showed multiple T2/FLAIR high-intensity lesions in white matter and slightly enlarged ventricles (Fig. 1A–D and I). MRI showed no morphological abnormalities in the corpus callosum or brainstem (Fig. 1J). At 14 years of age, physical descriptions of large ears, deep-set eyes, and a wide-based gait were recorded, but no photographs were taken (the parents did not consent to facial photographs). At 16 years of age, his body weight was 41.5 kg (−2.0 SD), his body height was 162.7 cm (−1.3 SD), and his head circumference was 51.8 cm (−3.2 SD). The T2/FLAIR high-intensity lesion on MRI gradually decreased (Fig. 1E–H).

**Fig. 1:**
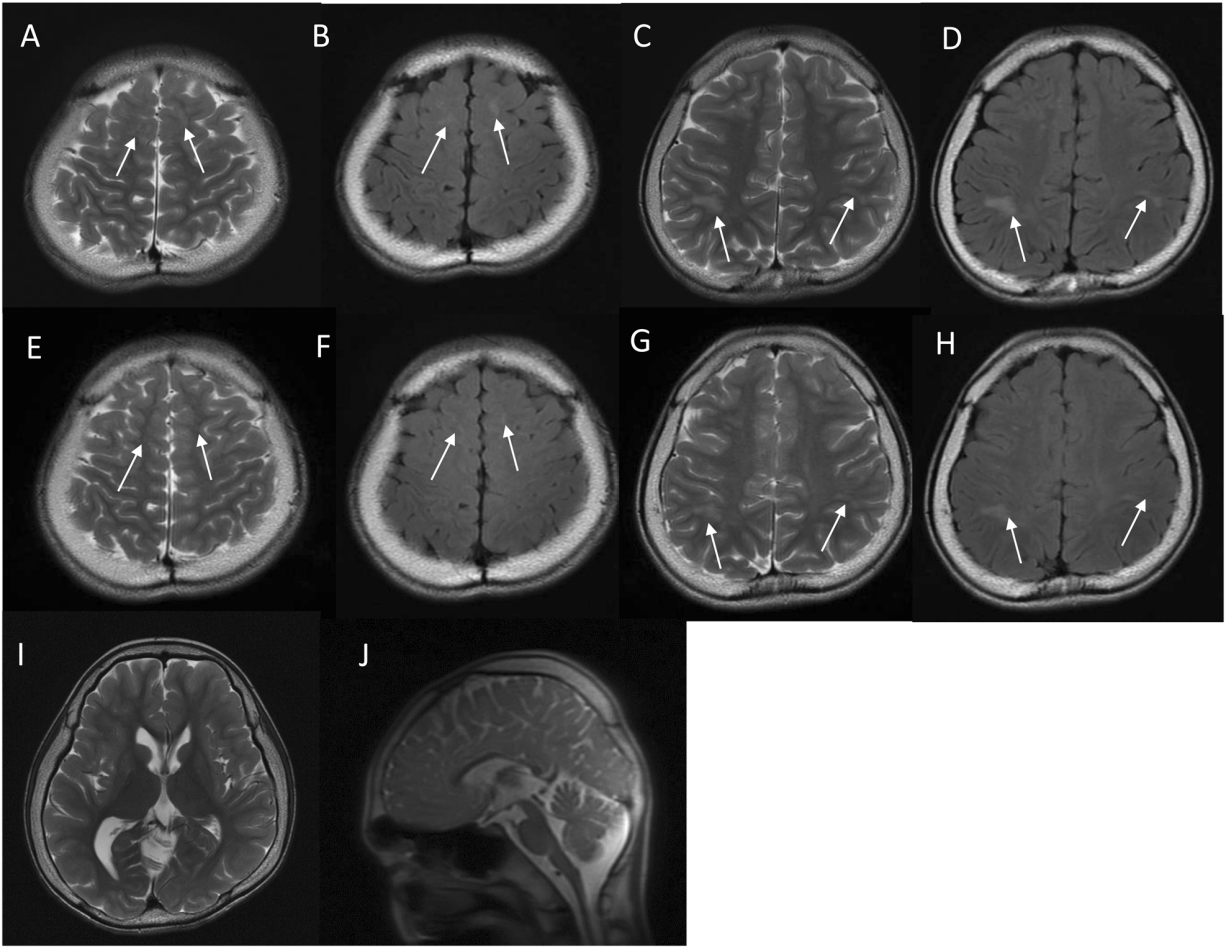
Brain magnetic resonance imaging (MRI) findings. Neuroimaging of the patient at **A**–**D** and **I**, **J** 11 years of age and **E**–**H** 15 years of age. **A**, **C**, **E**, **G**, **I** and **J** T2-weighted magnetic resonance imaging. **B**, **D**, **F**, and **H** Axial fluid-attenuated inversion recovery (FLAIR) magnetic resonance imaging. **A**–**D** Axial T2/FLAIR images showing hyperintense signals in white matter within the frontal and parietal lobes (arrows). The U-fibers were spared. **I** Axial T2-weighted imaging showed a slight enlargement of the left ventricle. **J** Sagittal T2-weighted imaging showed no morphological abnormalities in the corpus callosum or brainstem. **E**–**H** As in the image taken at 11 years of age, T2/FLAIR imaging showed hyperintense signals in white matter within the frontal and parietal lobes (arrows). The signal intensity of each lesion was lower in this image than in the one taken at 11 years of age.

After written informed consent was obtained from the parents, whole-exome sequencing was performed on them and the proband as previously described to determine the causative gene [4]. The examination protocols were approved by the Central Ethics Committee of Tohoku University School of Medicine Hospital (2018–2-216). The patient had a de novo heterozygous nonsense variant of the *DYRK1A* gene, NM_001396.4: c.957C>G, p.Tyr319*. This was confirmed using Sanger sequencing (Supplementary Fig. 1). This variant is novel, but the same amino acid change (c.957C>A, p.Tyr319*) has previously been reported in *DYRK1A*-related intellectual disability syndrome [5]. Based on the ACMG/AMP guidelines, this variant was rated as pathogenic [6].

Our patient’s clinical manifestations, consisting of physical and facial features as well as developmental and neuropsychiatric features, were compatible with the clinical manifestations observed in patients with *DYRK1A*-related intellectual disability syndrome [1, 6, 7]. The prevalence of epilepsy without febrile seizure was 33% in a previous study involving 15 patients [8]. Table 1 shows previous epilepsy cases with *DYRK1A*-related intellectual disability syndrome [7–9]. Regarding antiepileptic drugs, three cases were treated with valproate, and one case was treated with levetiracetam. As shown in Table 1, the EEG characteristics in these cases were unknown, and the seizure phenotypes of the two cases consisted of generalized seizures. As for the phenotype of the present case, the common seizure semiology of this disorder may be phenotyped as generalized seizures. The clinical course of epilepsy in patients with *DYRK1A*-related intellectual disability syndrome may be well controlled [7].

**Table 1:** Characteristics of epilepsy patients with *DYRK1A*-related intellectual disability syndrome.

	Patient 2 [7]	UMCN1 [8]	GF2852 [8]	.Moller et al. [9]	Present case
Genotype	c.787C>T	c.799C>T	c.367C>T	t(2,21)(q22;q22)	c.692G>A
Gender	F	F	M	F	M
Age (year)	12	59	32	13	18
Epilepsy onset age	1 year	N.D	2-4 years	1 year	1 year
Seizure semiology	Generalized tonic-clonic	Atonic and absences	N.D	Generalized tonic-clonic	Generalized atonic
AED	LEV	VPA	VPA	VPA, ESM	VPA
Seizure control	Well	Well	N.D	Not tolerated	Well
DQ/IQ	N.D	N.D	N.D	N.D	12
MRI_white matter abnormality	−	N.D	N.D	−	+
Meaningful words	+	−	+	−	−
Height	+2 SD	< −2.5 SD	−1.8 SD	−1.5 SD	−1.3 SD
Head	−1.3 SD	< −2.5 SD	< −2.5 SD	< −3 SD	−3.2 SD
Weight	+1.7 SD	+ 2 SD	−2.5 SD	−3 SD	−2.0 SD
Facial gestaut	+	+	+	+	+

*F* female, *M* male, *N.D* no data, *AED* antiepileptic drug, *VPA* valproate, *LEV* levetiracetam, *DQ* developmental quotient, *IQ* intellectual quotient, *SD* standard deviation.

Ventricular enlargement has already been reported in a patient with *DYRK1A*-related intellectual disability syndrome [1, 9]. In follow-up MRIs of the present patient, the white matter lesions had slightly reduced signal intensity compared to the first image. Ji J et al. described similar white matter lesions as gliosis [1]. *DYRK1A* has been noted to play an important role in neurogenesis, and white matter lesions in patients with pathogenic variants of *DYRK1A* may arise during the early development of the brain. However, such white matter lesions can also be caused by other factors that are not known to be associated with this gene, such as inflammation, vascular abnormality, and infection. The pathogenesis of white matter lesions in patients with pathogenic *DYRK1A* variants is unknown. The correlation between genotype and white matter abnormality is unknown in this disorder, and case with a different variant causing the same amino acid change did not show white matter abnormality [5].

## Supplementary information

Supplementary figure 1

## Data Availability

The relevant data from this Data Report are hosted at the Human Genome Variation Database at 10.6084/m9.figshare.hgv.3033.

## References

[1] Ji J (2015). DYRK1A haploinsufficiency causes a new recognizable syndrome with microcephaly, intellectual disability, speech impairment, and distinct facies. Eur. J. Hum. Genet.

[2] Blackburn ATM (2019). DYRK1A-related intellectual disability: a syndrome associated with congenital anomalies of the kidney and urinary tract. Genet. Med.

[3] O’Roak BJ (2012). Multiplex targeted sequencing identifies recurrently mutated genes in autism spectrum disorders. Science.

[4] Aoi H., et al. Whole exome sequencing of fetal structural anomalies detected by ultrasonography. *J. Hum. Genet*. 10.1038/s10038-020-00869-8 (2020). 10.1038/s10038-020-00869-8 33144663

[5] Qiao F (2019). A de novo mutation in DYRK1A causes syndromic intellectual disability: a Chinese case report. Front. Genet..

[6] Richards S (2015). ACMG Laboratory quality assurance committee. standards and guidelines for the interpretation of sequence variants: a joint consensus recommendation of the American College of Medical Genetics and Genomics and the Association for Molecular Pathology. Genet. Med.

[7] Bronicki LM (2015). Ten new cases further delineate the syndromic intellectual disability phenotype caused by mutations in DYRK1A. Eur. J. Hum. Genet.

[8] van Bon BW (2016). Disruptive de novo mutations of DYRK1A lead to a syndromic form of autism and ID. Mol. Psychiatry.

[9] Møller RS (2008). Truncation of the Down syndrome candidate gene DYRK1A in two unrelated patients with microcephaly. Am. J. Hum. Genet.

