# Supplementary material for: Clinical course of epilepsy and white matter abnormality linked to a novel DYRK1A variant
Source: Hum Genome Var. 2021 Jul 12;8:26. doi: 10.1038/s41439-021-00157-7 (PMC8275604; doi:10.1038/s41439-021-00157-7)
Supplement: Supplementary file 1 — Supplementary figure 1 [file 41439_2021_157_MOESM1_ESM.pptx]

## Slide 1
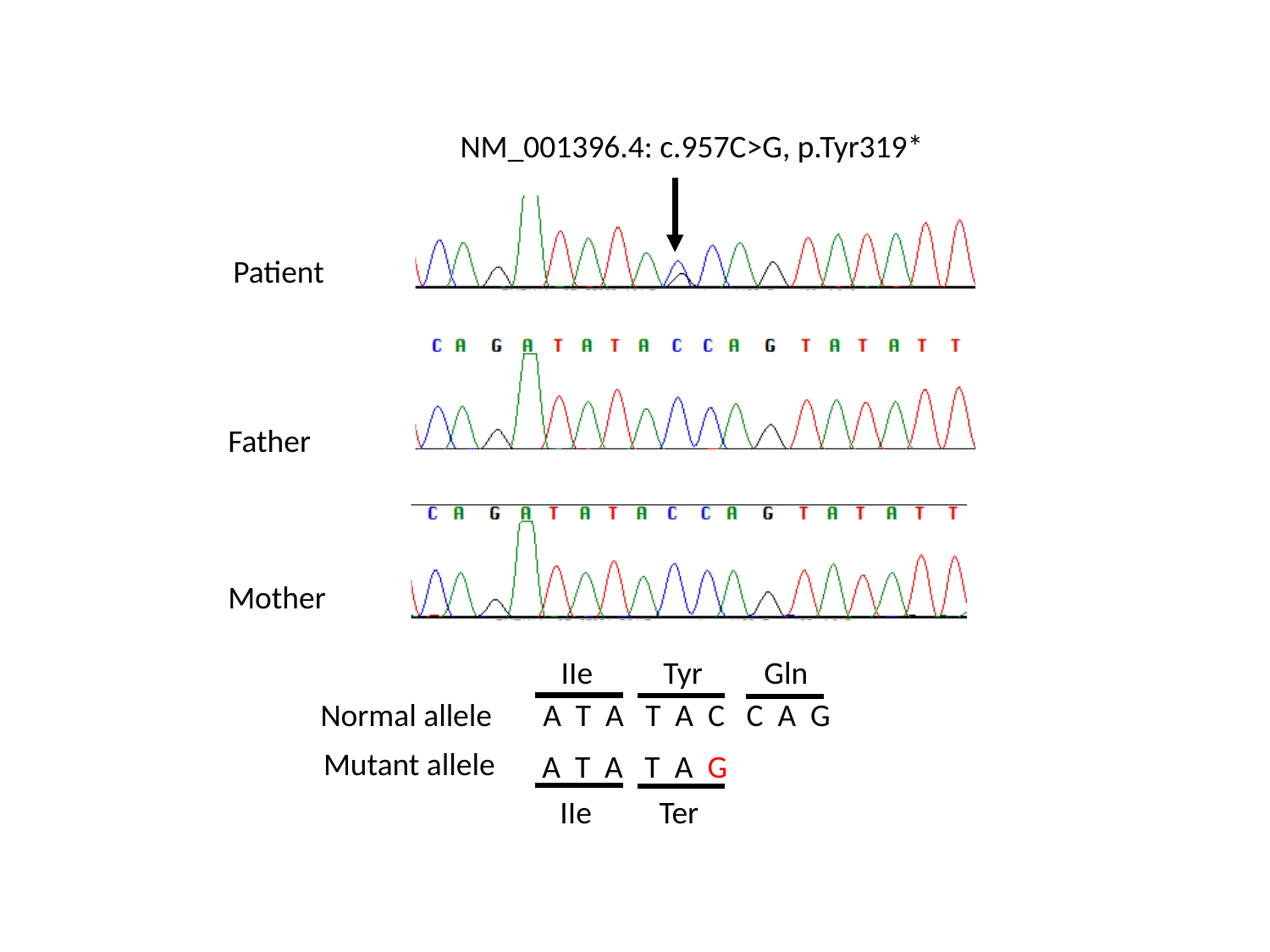

NM_001396.4: c.957C>G, p.Tyr319*
Patient
Father
Mother
IIe
Tyr
Gln
A T A T A C C A G
Normal allele
Mutant allele
A T A T A G
IIe
Ter
